# Wheat germ agglutinin-induced paraptosis-like cell death and protective autophagy is mediated by autophagy-linked FYVE inhibition

**DOI:** 10.18632/oncotarget.20436

**Published:** 2017-08-24

**Authors:** Tsung Lin Tsai, Hao Chen Wang, Chun Hua Hung, Peng Chan Lin, Yi San Lee, Helen H.W. Chen, Wu Chou Su

**Affiliations:** ^1^ Department of Internal Medicine, National Cheng Kung University Hospital, College of Medicine, National Cheng Kung University, Tainan, Taiwan; ^2^ Insititue of Clinical Medicine, College of Medicine, National Cheng Kung University, Tainan, Taiwan; ^3^ Department of Radiation Oncology, National Cheng Kung University Hospital, College of Medicine, National Cheng Kung University, Tainan, Taiwan

**Keywords:** cell death, wheat germ agglutinin, paraptosis, cytoplasmic vacuolation, Alfy

## Abstract

Wheat germ agglutinin (WGA) is a lectin that specifically binds cell surface glycoproteins and disrupts nuclear pore complex function through its interaction with POM121. Our data indicate WGA induces paraptosis-like cell death without caspase activation. We observed the main features of paraptosis, including cytoplasmic vacuolation, endoplasmic reticulum dilation and increased ER stress, and the unfolded protein response in WGA-treated cervical carcinoma cells. Conversion of microtubule-associated protein I light chain 3 (LC3-I) into LC3-II and punctuate formation suggestive of autophagy were observed in WGA-treated cells. WGA-induced autophagy antagonized paraptosis in HeLa and CaSKi cells, which expressed autophagy-linked FYVE (Alfy) protein, but not in SiHa cells that did not express Alfy. Alfy knockdown in HeLa cells induced paraptosis-like cell death. These data indicate that WGA-induced cell death occurs through paraptosis and that autophagy may exert a protective effect. WGA treatment and Alfy inhibition could be an effective therapeutic strategy for apoptosis-resistant cervical cancer cells.

## INTRODUCTION

Wheat germ agglutinin (WGA) is lectin consisting of two identical subunits. It inhibits export of nuclear RNA by the nuclear pore complex and protein shuttling through its interaction with POM121 [1–3]. WGA is suitable for targeted drug delivery because it binds specifically to membrane-associated glycoproteins that are highly expressed on the surfaces of some cancer cells [4–6]. Plant lectins have been investigated for the treatment of various tumors [7–9] and inflammatory diseases [10]. We previously demonstrated that WGA has a tumor-suppressive effect similar to that of nuclear signaling peptide-modified gold nanoparticles, which block nucleocytoplasmic transport [11]. However, the mechanisms underlying the anti-tumor activity of WGA have not been elucidated.

Cell death is classified as apoptosis, autophagy, necrosis, and cornification. Atypical mechanisms of cell death include paraptosis, mitotic catastrophe, anoikis, excitotoxicity, Wallerian degeneration, pyronecrosis, pyroptosis, entosis, and postface [12]. Apoptosis, autophagy, and paraptosis exhibit morphological features of programmed cell death (PCD) [13]. Apoptosis (type 1 PCD) is a caspase-dependent process characterized by chromatin condensation, nuclear fragmentation, cell shrinkage, plasma membrane blebbing, and apoptotic body formation [13]. In contrast, autophagy (type 2 PCD) is a dynamic process in which cytoplasmic proteins are sequestered and organelles become damaged forming double membrane vacuoles known as autophagosomes. The autophagy protein microtubule-associated protein 1 light chain 3 (LC3) participates in the fusion of autophagosomes and lysosomes. Autophagy has many crucial physiological functions including triggering lysosome-dependent degradation, organelle removal, and protein turnover, [14, 15]. Paraptosis (type 3 PCD) describes a form of PCD that is morphologically and biochemically distinct from apoptosis. It is characterized by cytoplasmic vacuolization originating from the swelling/dilation of the endoplasmic reticulum (ER) and/or mitochondria, and is devoid of any other morphological features of apoptosis [16, 17]. Caspase inhibitors typically cannot block paraptosis because it is not involved in caspase activation or the formation of apoptotic bodies. Cancer therapies that induce paraptosis may suppress the multi-drug resistant phenotypes often associated with resistance to apoptosis [18].

Autophagy-linked FYVE (Alfy/WDFY3) is a large, multi-domain scaffolding protein implicated in the selective degradation of ubiquitinated protein aggregates during autophagy [19]. Alfy predominantly localizes to the nucleus and nuclear membrane under basal conditions, but it can be recruited to ubiquitin-positive protein aggregates in the cytoplasm in response to stress [20]. Overexpression of Alfy decreased the number of protein inclusions and protected cells from expanded polyglutamine toxicity in an autophagy-dependent manner in a *Drosophila* eye model of Huntington disease [21]. Alfy also promoted the autophagic removal of misfolded proteins involved in amyotrophic lateral sclerosis suggesting it may be a useful target for the treatment of this disease [22]. Low levels of Alfy lead to increased ER stress and the aggregation of p62-positive polyubiquitinated proteins, which promoted autophagy in rheumatoid arthritis synovial fibroblasts [23].

We investigated the mechanisms underlying WGA-induced cell death in cervical carcinoma cells by assessing cytotoxicity, cytoplasmic vacuolation, and caspase activation in WGA-treated in HeLa, SiHa, and CaSKi cells. Additionally, we analyzed the role of Alfy in the ER stress response, cellular vacuolation, and cell death in WGA-treated cells.

## RESULTS

### WGA induces cytoplasmic vacuolization and cell death in cervical carcinoma cells

**Figure 1:**
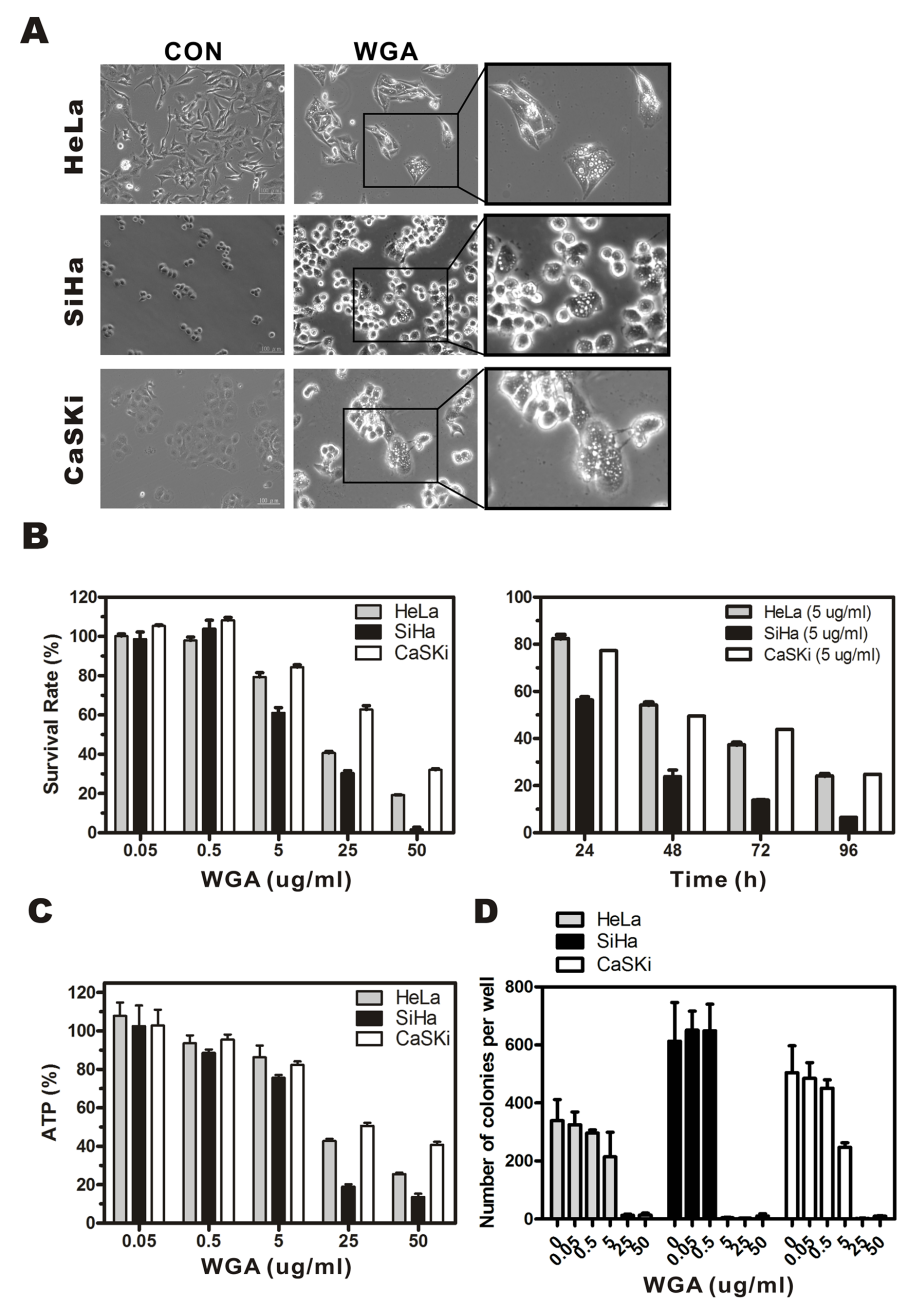
WGA induces formation of cytoplasmic vacuoles and paraptosis-like cell death in cervical carcinoma cells **(A)** Light micrographs of WGA-treated and untreated control cancer cells. HeLa cells, SiHa cells, and CaSKi cells were treated with WGA (10, 5, and 20 μg/mL, respectively). **(B)** MTT assays of cell viability. Cells were incubated with WGA at different concentrations for 24 h, or with a fixed concentration of 5 μg/mL for different lengths of time. **(C)** ATP levels 24 h after treatment with WGA at the indicated concentrations. **(D)** Clonogenic assays showing decreased viability of HeLa, SiHa, and CaSKi cells after treatment with WGA at concentrations ranging from 0.05–50 μg/mL. After long-term incubation (10–14 days), cells were fixed and stained with crystal violet, and the number of colonies counted. Data are expressed as the mean ± SD based on three independent experiments.

We performed MTT assay to assess the cytotoxicity of WGA in HeLa, SiHa, and CaSKi cervical carcinoma cell lines. Extensive vacuolization was observed in HeLa, SiHa, and CaSKi cells by light microscopy 24 h after WGA treatment (Figure 1A). Cells with vacuoles surrounding the nuclei had detached from the plates. WGA treatment resulted in a dose- and time-dependent reduction in cell viability. The IC_50_ of WGA after 24 h was approximately 20.4 μg/mL for HeLa cells, 12.3 μg/mL for SiHa cells, and 31.9 μg/mL for CaSKi cells. Cell death gradually increased between 24 h and 96 h at a rate of 17.6% ± 3.0% to 75.8% ± 1.5% in HeLa cells, 43.6% ± 2.6% to 93.5% ± 0.2% in SiHa cells, and 22.7% ± 2.7% to 75.1% ± 3.7% in CaSKi cells (Figure 1B). Cell viability was assessed using an ATP bioluminescence assay. ATP levels gradually decreased in WGA-treated cells compared to untreated control cells (Figure 1C). ATP levels decreased from 107.9% ± 12.1% to 25.6% ± 1.4% in HeLa cells, 102.6% ± 21.5% to 13.6% ± 3.6% in SiHa cells, and 102.9% ± 16.4% to 40.8% ± 3.1% in CaSKi cells. Sustained WGA treatment for 14 days resulted in increased cell death, indicating WGA was a potent inhibitor of cervical carcinoma cell growth (Figure 1D).

### WGA induces paraptosis and autophagy in HeLa and CaSKi cells, and paraptosis in SiHa cells

**Figure 2:**
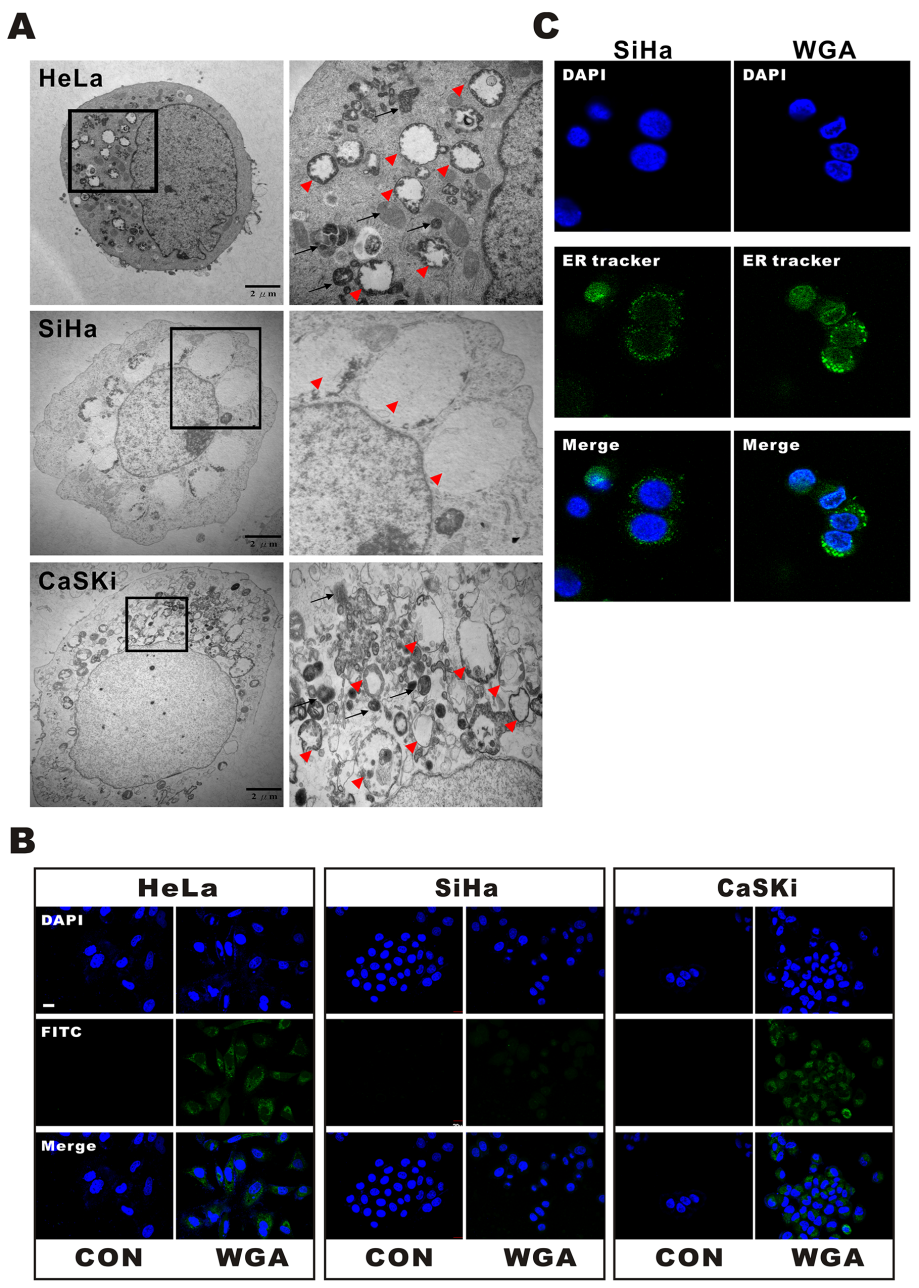
WGA induces both paraptosis and autophagy in HeLa and CaSKi cells, but only paraptosis in SiHa cells **(A)** TEM images of HeLa, SiHa, and CaSKi cells treated with WGA for 24 h. The inset shows a part of the image at higher magnification. The red arrow heads point to extensive, clear paraptosis vacuoles. The black arrows point to autophagosomes or autolysosomes. **(B)** Autophagic vacuoles are stained with Cyto-ID^®^ green fluorescent dyeandthenuclei with DAPI. An increase in green fluorescence was observed in WGA-treated HeLa and CaSKi cells. Little to no staining of vacuoles was observed in SiHa or control cells. **(C)** Confocal images of SiHa cells with and without WGA treatment for 24 h. The ER structures are stained with ER-tracker and the nuclei with DAPI.

WGA-induced cytoplasmic vacuolization was visualized by transmission electron microcopy (TEM) (Figure 2A). WGA-treated cells exhibited two types of cytoplasmic vacuolization. Small vacuoles containing cytoplasmic organelles such as mitochondria and ER (high density in vacuoles) were engulfed by multi-membrane structures in WGA-treated HeLa and CaSKi cells (Figure 2A, black arrow), suggesting the existence of autophagosomes or autolysosomes. Other vacuoles were more extensive and clear of protein material (Figure 2A, red arrow head). Interestingly, only WGA-treated SiHa cells had extensive and clear vacuoles, suggesting that cell death may have resulted from a different mechanism in these cells.

We next investigated whether the vacuoles were involved in autophagy. WGA-treated cells were harvested and stained with Cyto-ID™, a fluorescent probe for rapidly tracking vacuoles and autophagy in living cells. Treatment of HeLa and CaSKi cells with WGA for 24 h resulted in the accumulation of lysosomal vacuoles with autolysosomal characteristics. However, only slight enhancement of fluorescence was observed in SiHa cells (Figure 2B). No vacuoles were observed in control cells. We added an ER-tracker stain to SiHa cells to visualize the morphological dynamics of cytoplasmic vacuolization (Figure 2C). Cytoplasmic vacuolization and ER dilation are typical features of paraptosis [16].

### WGA induces non-apoptotic cell death

**Figure 3:**
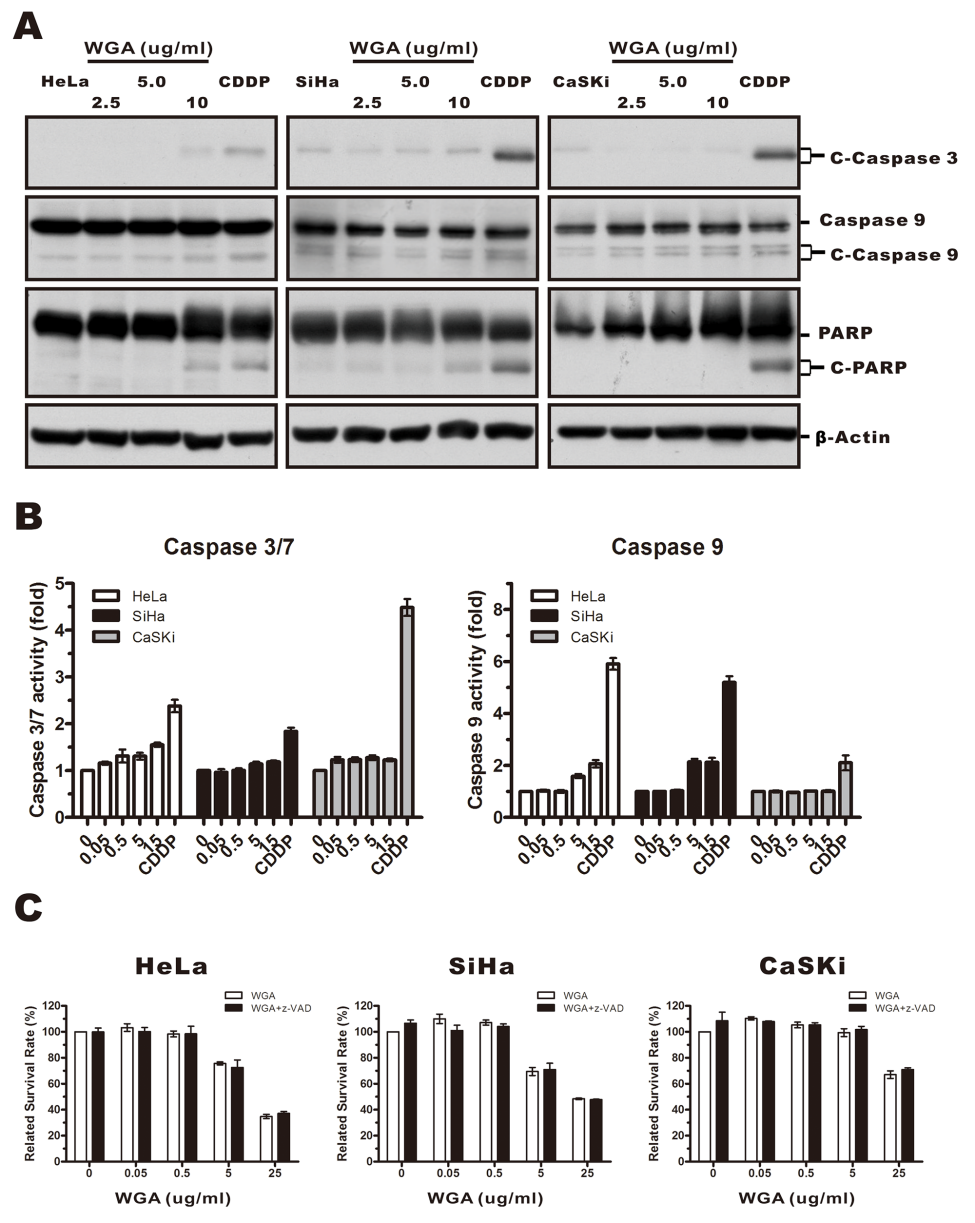
WGA-induced cytoplasmic vacuolation and cell death was not associated with caspase-dependent apoptosis **(A)** HeLa, SiHa, and CaSKi cells were treated with 2.5, 5, or 10 μg/mL WGA for 24 h, and the expression of caspase-3, -9, and PARP measured by western blot. β-Actin was used as a loading control. **(B)** The activity of caspase-3/7 and caspase-9 was detected using Caspase-Glo assays. Data are expressed as the mean ± SD for four replicates. **(C)** HeLa, SiHa, and CaSKi cells (untreated or pre-treated with 25 μM z-VAD-fmk) were treated with WGA at the indicated concentrations. Cell survival was assessed using MTT assays. Data are expressed the mean ± SD for six duplicated experiments.

Because paraptosis typically does not involve caspase activation, apoptotic body formation, or DNA fragmentation [24], we investigated whether caspases were activated in response to WGA treatment. We measured caspase-3, -9, and poly-ADP-ribose polymerase (PARP) protein levels in WGA- compared to cisplatin-treated cells using antibodies that specifically recognized the cleaved forms of caspase-3, -9, and PARP. Caspase-3 and -9 levels were unchanged while the levels of PARP slightly increased (Figure 3A). We confirmed these results using Caspase-Glo assays (Figure 3B). No difference in the percentage of dead cells was observed between cells pre-treated with 20 μM of z-VAD-fmk (a broad spectrum pan-caspase inhibitor) followed by WGA compared to untreated control cells (Figure 3C). Thus, WGA-induced cell death likely resulted from paraptosis.

### WGA induces autophagy and antagonizes paraptosis-like cell death in HeLa and CaSKi cells, but not in SiHa cells

**Figure 4:**
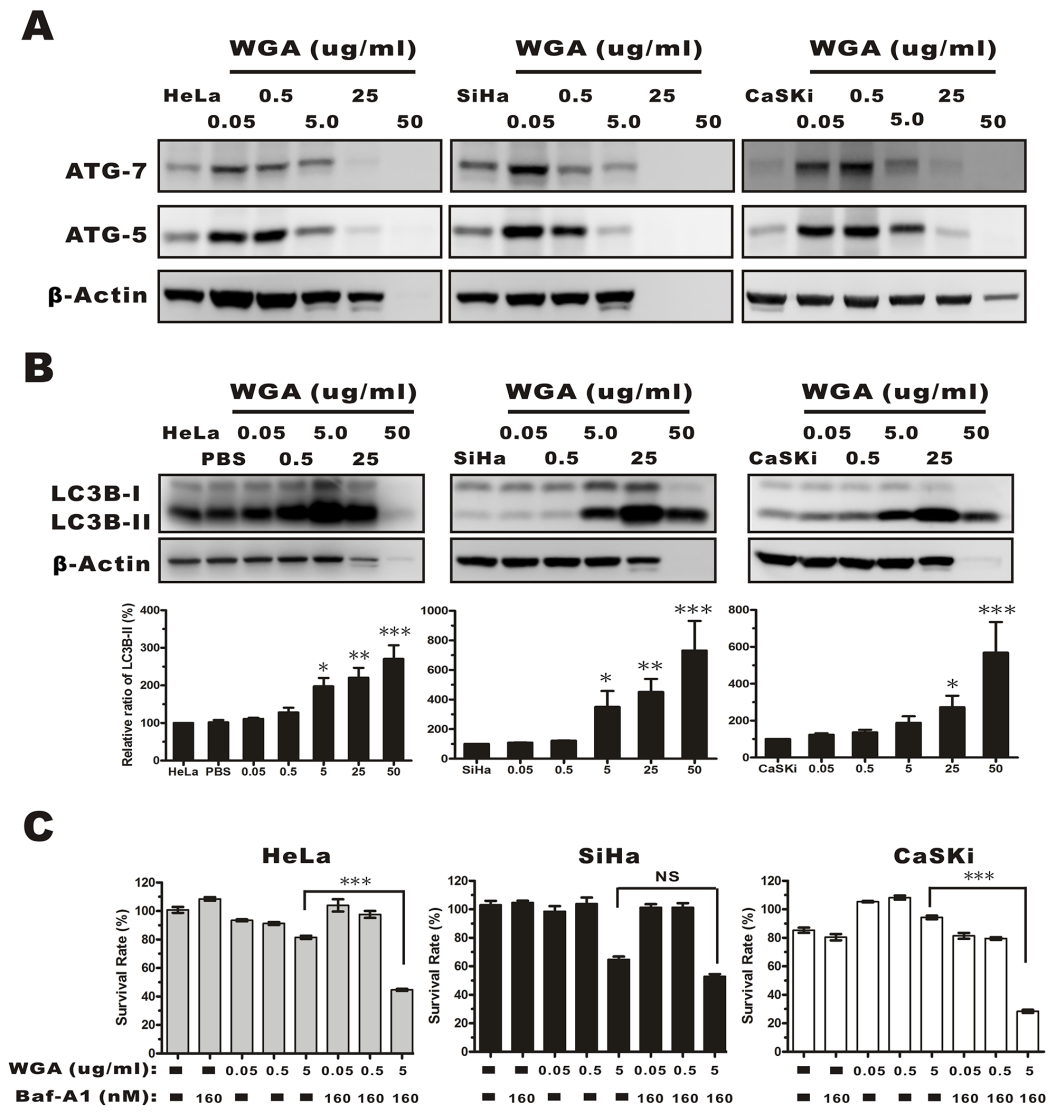
Evaluation of autophagy by monitoring ATG-5, -7, and the conversion LC3B-I to LC3B-II in whole protein extracts from HeLa, SiHa, and CaSKi cells treated with WGA at the indicated concentrations **(A)** ATG-5 and -7 expression increased in all cervical carcinoma cells following treatment with WGA at the indicated concentrations. **(B)** Western blot showing dose-dependent expression of LC3B in HeLa, SiHa, and CaSKi cells treated with WGA for 24 h. The cytoplasmic form of LC3 (LC3B-I) and the autophagosomal membrane-bound form (LC3B-II) were both detected. We quantified the relative levels of LC3-II to β-Actin. Bars represent the mean ± SD of three independent experiments (*, P < 0.05; **, P < 0.01; ***, P < 0.001). **(C)** MTT assays in HeLa, SiHa, and CaSKi cells treated with the indicated concentrations of WGA in the presence and absence of Baf-A1, an inhibitor of autophagy. Bars represent the mean ± SD of four independent experiments (P > 0.05, not significant; ***, P < 0.001).

We next investigated the effects of WGA on autophagy. The expression of autophagy-related genes (ATG) 5 and 7, and LC3B was measured by western blotting. ATG 5 and ATG 7 expression gradually increased in HeLa, SiHa, and CaSKi compared to control cells following treatment with 0.05 μg/mL WGA (Figure 4A). Once autophagy is activated, pro-LC3 is cleaved at the C-terminus to form LC3A, and modified by lipidation to generate LC3B, which can be incorporated into autophagosome membranes [25]. LC3B expression is correlated with autophagy progression and can be measured by calculating the percentage of membrane-bound 16 kDa protein (LC3B-II) relative to the percentage of β-actin. We measured LC3B-II levels in all three cervical carcinoma cell lines. The levels were upregulated in a dose-dependent manner in all WGA-treated cells (0.5–25 μg/mL) (Figure 4B). We investigated whether WGA-induced autophagy led to cell survival or death. Cells were treated with various concentrations of WGA. A fraction of the cells were pre-treated with 160 nM bafilomycin A1 (Baf-A1, an inhibitor of autophagy that interferes with the fusion of the autophagosome and lysosome). We observed a decrease in cell survival in Baf-A1-treated HeLa (81.53% ± 1.95% vs. 44.62% ± 1.36%) and CaSKi cells (94.33% ± 2.52% vs. 28.37% ± 2.99%), but not in Baf-A1-treated SiHa cells (61.07% ± 5.29% vs. 54.6% ± 3.84%) (Figure 4C). These data suggested that autophagy protected cervical carcinoma cells from WGA-induced paraptosis-like cell death.

### WGA promotes ER stress and the unfolded protein response

**Figure 5:**
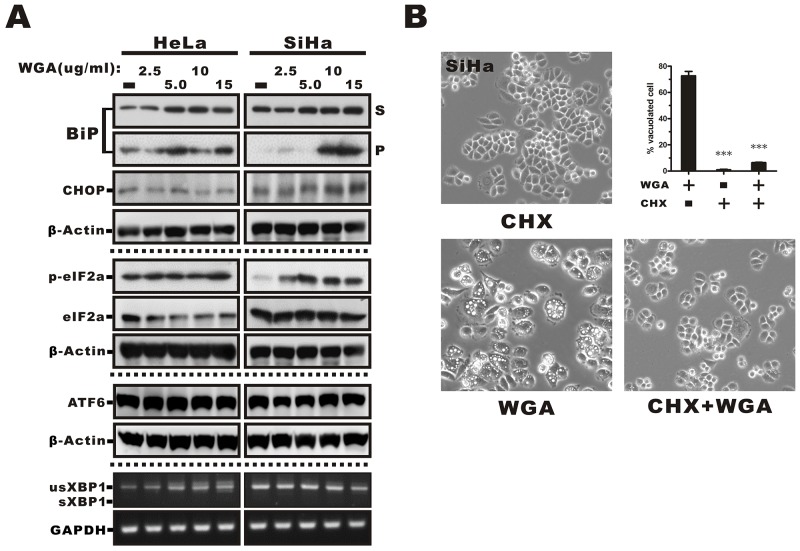
ER stress-mediated activation of the UPR in WGA-treated cervical carcinoma cells and prevention of vacuolation by CHX **(A)** Western blot analysis showing increased expression of markers of ER stress, including phosphorylated eIF2α, BiP, and ER chaperones in the Triton-soluble (S) or -insoluble pellet fractions (P), and the transcription factor CHOP in WGA-treated cancer cells. No changes in ATF6 expression or XBP1 splicing were observed in response to WGA treatment. **(B)** Inhibition of cytoplasmic vacuolation after the addition of CHX. SiHa cells were treated with either WGA (5 μg/mL) alone or in combination with CHX (25 μM) for 1.5 h and the percentage of vacuolated cells determined by counting at least 200 cells in three independent experiments. Bars represent the mean ± SD (***, P < 0.001).

ER stress and the unfolded protein response (UPR) trigger paraptosis [26]. We observed dilation of ER structures in response to WGA treatment, suggesting that vacuolization might occur as a result of ER stress. Therefore, we measured the effects of WGA treatment on ER resident proteins, which are known to be involved in protein folding, as an indicator of ER stress and UPR activation. The ER stress marker GRP78/BiP, a lumenal ER chaperone commonly associated with misfolded protein aggregates in the ER in response to external stimulation, is detectable in detergent (e.g. Trition X-100) insoluble cellular fractions [27]. We analyzed the distribution of BiP in Triton-soluble and Triton-insoluble fractions from WGA-treated cells. BiP was detected in both fractions in HeLa cells and exhibited dose-dependent upregulation (52% to 71%) in the Triton-soluble fraction. It was predominantly detected in the Triton-soluble fraction in SiHa cells. However, WGA treatment caused migration of BiP into the Triton-insoluble pellet fraction (P, Figure 5A). We also measured the expression of the C/EBP homologous protein (CHOP) and found that it was expressed in SiHa but not HeLa cells. Similarly, there was higher expression of phosphorylated eukaryotic initiation factor 2α (eIF2α) in SiHa compared to HeLa cells, which suggested that the UPR was activated in WGA-treated cells. No changes in ATF6 or XBP1 expression (and splicing) were observed (Figure 5A).

### WGA-induced vacuolation is reversed by cycloheximide treatment

Because an overload of protein in the ER lumen can lead to dilation, we hypothesized that cell death could be prevented by inhibiting protein synthesis. Pre-treatment of SiHa cells with cycloheximide (CHX), an inhibitor of eukaryote protein synthesis, blocked WGA-induced cytoplasmic vacuolation (Figure 5B). Thus, WGA induced active ER protein loading during vacuolation in a proteasome-dependent process was replaced by a protein synthesis dependent process which will be more suitable for the description of this result.

### Alfy knockdown conferred protection against WGA-induced cytoplasmic vacuolation and cell death

**Figure 6:**
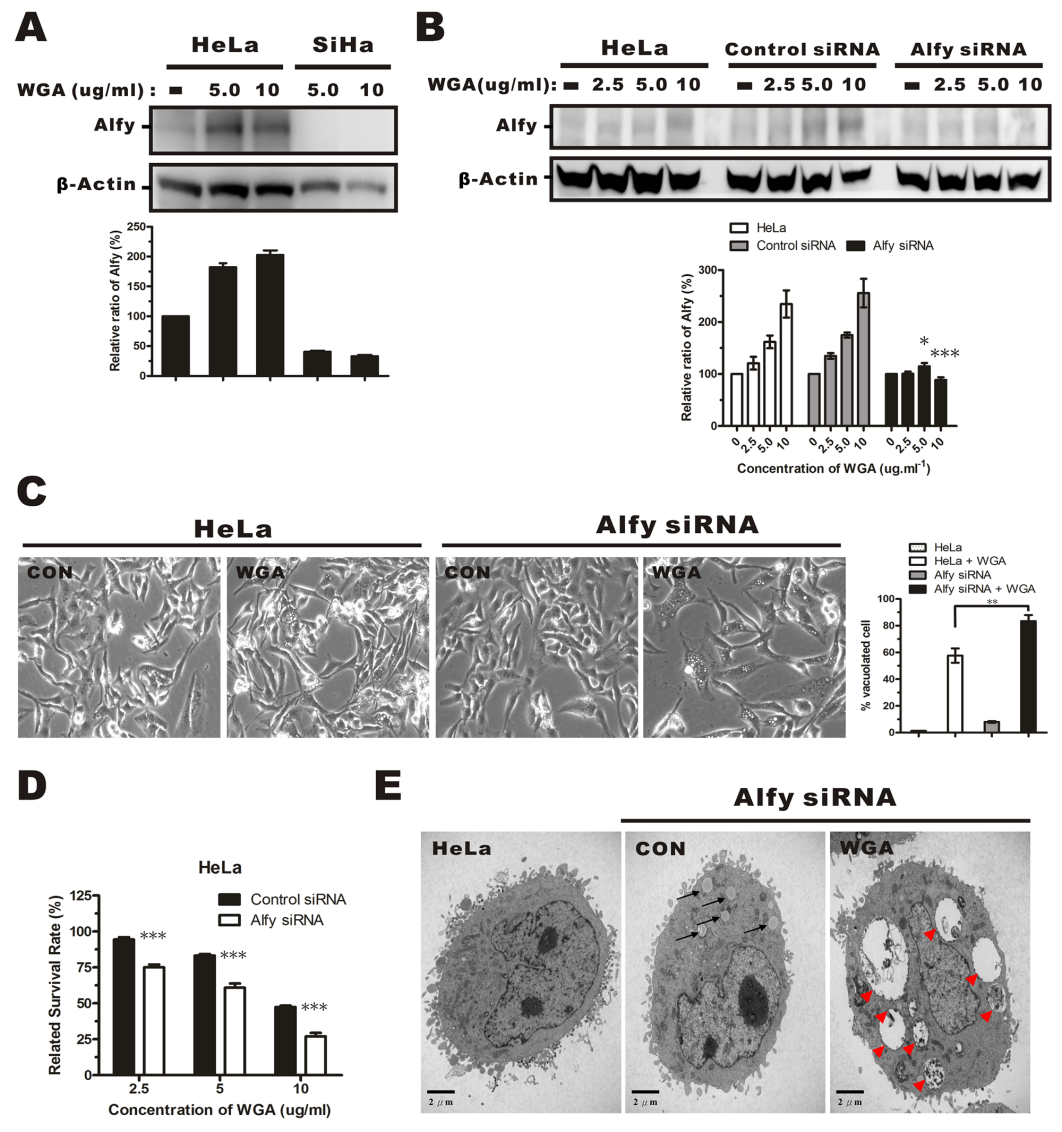
Effects of Alfy knockdown on WGA-induced cytoplasmic vacuolation and cell death **(A)** Western blotsof total cell lysates showing increased Alfy expression in HeLa cells, but not SiHa cells, after treatment with WGA (5.0 and 10 μg/mL) for 24 h. **(B)** Western blot showing Alfy expression in WGA-treated HeLa cells following knockdown of Alfy with shRNA. Empty vector (control siRNA) was used as negative control. The fold change in Alfy expression after WGA treatment was quantified using NIH ImageJ. The relative units were normalized to β-Actin and compared to untreated control cells. Data are expressed as the mean ± SD for three independent experiments. (*, P < 0.05; ***, P < 0.001) **(C)** Phase-contrast images showing the effects of Alfy knockdown on WGA-induced cytoplasmic vacuolation. Bar graph showing the percentage of vacuolated HeLa cells expressing control siRNA or Alfy siRNA with or without WGA treatment. At least 200 cells were counted in three independent experiments. Bars represent the mean ± SD (**, P < 0.01). **(D)** MTT assays of cell viability after treatment of Alfy knockdown and control HeLa cells with or without WGA for 24 h. Data represent an average of three independent experiments. Bars represent the mean ± SD (***, P < 0.001). **(E)** Formation of cytoplasmic vacuoles in HeLa cells following Alfy knockdown. Cells were transfected with Alfy siRNA and treated for 24 h with or without WGA (10 μg/mL). The black arrow head points to small vacuoles and the red arrow head points to extensive paraptosis-like vacuoles.

Alfy, an autophagy adaptor protein, has been shown to facilitate autophagic degradation of protein aggregates and reduce the number of protein inclusions to prevent cytotoxicity [22, 28]. We investigated whether WGA treatment altered Alfy expression in HeLa and SiHa cells. WGA treatment resulted in a dose-dependent increase in Alfy expression in HeLa cells. However, it barely induced expression of Alfy in SiHa cells (Figure 6A), suggesting that low Alfy expression might lead to reduced cellular survival in SiHa cells. We infected HeLa cells with lentiviral particles containing Alfy shRNA (Figure 6B). Knockdown of Alfy in WGA-treated cells led to an increase in vacuole formation (Figure 6C) and cell death (Figure 6D). Small vacuoles were visible by TEM in Alfy knockdown HeLa cells that were not treated with WGA (Figure 6E, black arrow). Similar vacuole morphology (extensive, clear, and lacking any visible cytoplasmic material) was observed in WGA-treated SiHa cells (Figure 6E, red arrow head). These data indicated that Alfy inhibited paraptosis in WGA-treated cells.

### Alfy knockdown enhances ER stress-induced cytoplasmic vacuolation and the UPR

**Figure 7:**
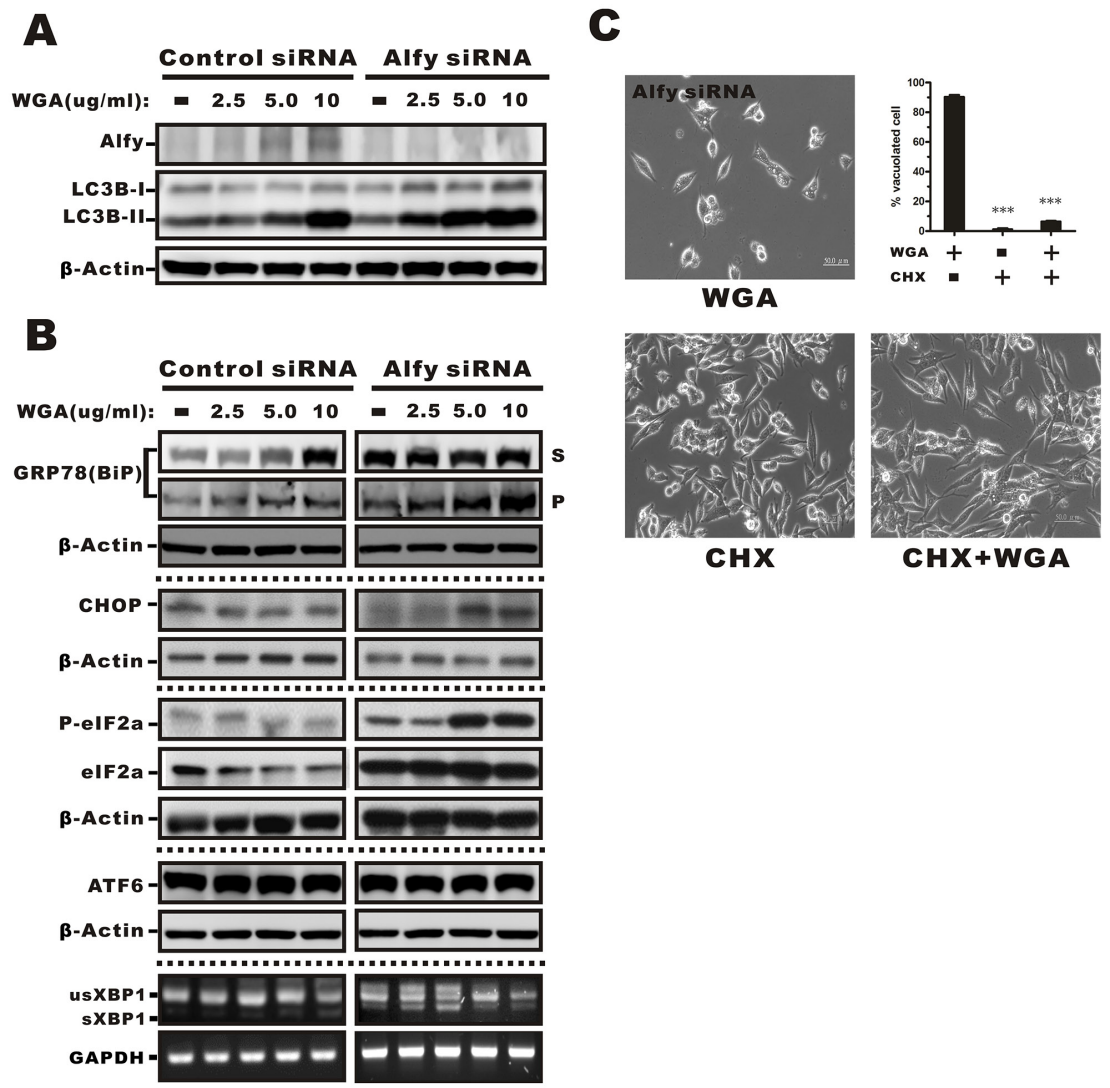
Effects of WGA on Alfy expression and the effects of Alfy knockdown ER stress-mediated cytoplasmic vacuolation and the UPR **(A)** Expression of Alfy andLC3 in HeLa cells in Alfy knockdown or vector control cells measured by Western blotting following treatment with the indicated concentrations of WGA. β-Actin was used as a loading control. **(B)** Markers of ER stress were evaluated in Alfy knockdown and vector control HeLa cells. Levels of phosphorylated eIF2α, BiP, ER chaperones, and CHOP in the Triton-soluble fraction (S) or -insoluble pellet (P) fraction in Alfy knockdown HeLa cells treated with WGA. No change in ATF6 expression or XBP1 splicing were observed in response to WGA treatment in Alfy knockdown or control HeLa cells. **(C)** Inhibition of cytoplasmic vacuolation in response to CHX. Alfy knockdown HeLa cells were treated with WGA (5 μg/mL) alone or in combination with CHX (25 μM) for 1.5 h and the percentage vacuolated cells calculated by counting at least 200 cells in three independent experiments. Bars represent the mean ± SD (***, P < 0.001).

Because WGA treatment resulted in increased ER stress and the UPR in SiHa cells that did not express Alfy, but only had a minor effect on these processes in HeLa cells that express Alfy, we investigated whether Alfy knockdown in HeLa cells would promote the ER stress response and vacuolation. We observed an increase in LC3 expression in HeLa cells infected with lentiviral particles containing Alfy shRNA and treated with various concentrations of WGA (Figure 7A). BiP, CHOP, and phosphorylated eIF2α levels were indicative of ER stress and activation of the UPR, but no changes in ATF6 or XBP1 mRNA splicing were observed (Figure 7B). CHX blocked WGA-induced cytoplasmic vacuolation in Alfy knockdown HeLa cells (Figure 7C), which was consistent with paraptosis [29]. Thus, knockdown of Alfy in HeLa cells followed by treatment with WGA resulted in a phenotype that resembled paraptosis in response to ER stress.

## DISCUSSION

WGA specifically binds to sugars on the surfaces of epithelial and immune cells. It can also induce apoptosis [7, 30–32]. WGA induced vacuolation, loss of cell architecture, and up-regulation of the apoptosis-related proteins Bax and caspase-3 was previously demonstrated in L929 cells. However, inter-nucleosomal DNA fragmentation, apoptotic bodies, and related apoptotic phenotypes were not detected [33]. Paraptosis does not exhibit the typical features of apoptosis. It is defined as cell death accompanied by massive cytoplasmic vacuolization as a result of a dilated ER lumen and the integrity of the plasma membrane both can be disrupted through inhibition of SAPK/JNK and activation of ERK-1/2 chemically or genetically [16, 34, 35]. We found that WGA induced caspase-independent paraptosis in cervical carcinoma cells. We also observed protective autophagy, which was more pronounced in cell lines that expressed Alfy.

WGA affected cell survival by increasing cytoplasmic vacuole enlargement. An extensive distribution of empty vacuoles consistent with paraptosis was observed in WGA-treated SiHa cells by TEM. Cytoplasmic material-containing vacuoles that are characteristic of autophagy were observed in HeLa and CaSKi cells only. These data indicated WGA induced autophagy in HeLa and CaSKi, but not SiHa cells. Previous studies have demonstrated that the amalgamation of ATG-5 to ATG-12 with the aid of ATG-7 plays a crucial role in autophagy [36]. We found that WGA induced expression of ATG-5 and -7 in all cell lines and a higher increase in LC3B-I to LC3B-II conversion in SiHa cells. Kar et al. [37] demonstrated that upregulation and processing of LC3 was an important event in non-autophagic cytoplasmic vacuolation and cell death. All three LC3 isoforms are required for the generation of autophagic vacuoles, but paraptosis only requires conversion of the B isoform of LC3 [38, 39]. Recent studies suggest that autophagy can protect against or induce PCD [40–42]. We found that inhibition of autophagic flux resulted in reduced cell viability in WGA-treated HeLa and CaSKi cells, but not SiHa cells (Figure 4C). Thus, autophagy may play a role in cell survival in response to WGA-induced paraptosis.

Alfy plays a role in the development of protein granules and autophagic membranes through its interaction with p62 [20; 28]. Under conditions of ER stress, there is an imbalance in the expression of Alfy and p62. Autophagy can lead to cell death, but it can also protect cells from MG132-induced apoptosis in an Alfy-dependent manner [23]. We have shown that WGA induces ER stress and the UPR as evidenced by activation of ER chaperones such as BiP and CHOP, elevated levels of phosphorylated eIF2α, and the prevention of cytoplasmic vacuolation by CHX, which halts protein translation and prevents the accumulation of misfolded proteins. Higher levels of BiP under conditions of ER stress were shown to prevent CHOP-mediated apoptosis [43]. We found that higher levels of BiP promoted paraptosis in SiHA cells. Therefore, the observed differences in cell viability in HeLa, SiHa, and CaSKi cells may have resulted from differences in Alfy expression at the time of WGA treatment.

We observed an association between autophagy and increased Alfy expression in WGA-treated HeLa cells, which conferred protection from cell death. WGA induced low Alfy expression in SiHa cells, which underwent cell death by paraptosis. Knockdown of Alfy in HeLa cells resulted in massive cytoplasmic vacuolation and decreased cell viability. The morphologies of these cells were similar to those of WGA-treated SiHa cells. These data indicate that Alfy levels regulate WGA-induced cell death and that Alfy acts as a switch that determines whether cells die through paraptosis or autophagy.

Increases in BiP, CHOP, phosphorylated eIF2α, and membrane-bound LC3-II, as well as massive cytoplasmic vacuolation, were observed in WGA-treated, Alfy knockdown cells. Similar alterations were observed in WGA-treated SiHa cells. Thus, WGA-induced paraptosis-like cell death may be associated with the induction and processing of LC3B [44]. Alfy induces autophagy to protect cells from WGA-induced paraptosis-like cell death.

Alfy knockdown resulted in an increase in the number of small vacuoles in HeLa cells in the absence of WGA-induced ER stress. Treatment of Alfy knockdown cells with WGA resulted in vacuoles that appeared more paraptosis-like (extensive, clear, and lacking any visible cytoplasmic material). Therefore, Alfy may regulate these alternative forms of paraptosis-associated vacuolization.

We have demonstrated that WGA induces ER stress and the UPR, resulting in paraptosis-like cell death. Alfy promoted protective autophagy in WGA-treated cells. WGA could potentially be used to target cancer cells such as SiHa cervical carcinoma cells that lack Alfy expression.

## MATERIALS AND METHODS

### Cell culture and reagents

Human cervical carcinoma HeLa and SiHa cells were cultured in Dulbecco Modified Eagle Medium (DMEM) containing 10% fetal bovine serum and 1% antibiotics (penicillin, streptomycin, and amphotericin B) at 37°C in a humidified 5% CO_2_ incubator. CaSKi cells were cultured in RPMI 1640 medium under the same conditions. When the cells reached 80% confluence, they were trypsinized and seeded into plates/dishes for experiments. WGA (SI-L0636), Z-VAD-FMK (V116), Baf-A1 (B1793), and cycloheximide (C4850) were purchased from Sigma-Aldrich (St. Louis, MO, USA). The rabbit anti-Alfy antibody (AV50850) was also obtained from Sigma-Aldrich. The rabbit anti-LC3B antibody, phosphorylated and total MAPK Family Antibody Sampler Kit, mouse anti-ubiquitin, rabbit anti-Bip, mouse-anti ubiquitin, rabbit anti-phosphorylated ERK and Akt, mouse anti-β-actin, and GAPDH antibodies were purchased from Cell Signaling Technology (Danvers, MA, USA). The ER-tracker was purchased from Invitrogen (Eugene, Oregon, USA).

### Cell viability assays and quantification of vacuolated cells

Cells were seeded in 96-well plates at a density of 10^3^ cells per well in complete medium and incubated overnight. A series of concentrations of WGA (0, 0.05, 0.5, 5, 25, and 50 μg/mL) were added and the cells incubated for 24 h. Alternatively, a fixed concentration of WGA (10 μM) was added and the cells incubated for different lengths of time (24, 48, 72, and 96 h). Cell viability was analyzed using MTT assays and the ATP Bioluminescence Assay Kit (Promega, Madison, WI, USA). Light micrographs were obtained for different fields of view, and vacuolated cells were counted (at least 150 cells per condition).

### Colony formation assays

HeLa, SiHa, and CaSki cells were seeded into six-well plates at a density of 10^3^ cells per well. After 24 h, the cells were treated with various concentrations of WGA for 14 days. The drug was replaced each time the medium was replaced. Plates were then fixed with 1% acetic acid in 50% methanol for 30 min, washed three times with PBS, and stained with 0.5% crystal violet (Sciencelab.com, Inc., Houston, TX, USA) in 70% methanol for 30 min. The plates were rinsed with distilled water until the background was clear. Images were captured using a Bio-Rad VersaDoc™ imaging system (Hercules, CA, USA) and colonies were counted.

### Transmission electron microscopy

The ultra-structure of cytoplasmic vacuoles was visualized using a JEOL-1200 Transmission Electron Microscope with an accelerating voltage of 80 kV. Cells were fixed with 2% paraformaldehyde and 2.5% glutaraldehyde for 30 min at room temperature, washed with PBS, and post-fixed with 1% osmium tetraoxide in 0.1 M Na-cacodylate buffer (pH 7.2) for 1 h. Samples were washed and dehydrated in graded concentrations of ethanol (50%, 70%, and 100%) and propylene oxide. They were then embedded in Epon resin (Fluka, Buchs, Switzerland) and cut into ultrathin sections. Sections (80 nm thick) were collected on copper TEM grids and stained with 5% uranyl acetate for 20 min and lead citrate for 10 min. Sections were then imaged by TEM.

### Cyto-ID^®^ green fluorescent dye staining for the detection of autophagy

Cells were stained with Cyto-ID^®^ green fluorescent dye (Enzol Life Sciences, Farmingdale, NY, USA) for the detection of autophagic vacuoles. Approximately 2 × 10^5^ cells were cultivated in 6-well chamber slides (BD, Bedford, MA, USA) with different concentrations of WGA (5 and 10 μg/mL) and dye for 24 h. Cells were fixed and the nuclei stained with 4',6-diamidino-2-phenylindole (DAPI). Fluorescent and cellular morphology images were obtained using an Olympus FluoView™ FV1000 fluorescence microscope (FV1000, Olympus, Japan).

### ER staining with ER-tracker

ER staining was performed with an ER-tracker kit (Invitrogen). Briefly, WGA-treated cells were washed with 1 x PBS and incubated in warmed ER-tracker dye solution (1 μM) for approximately 30 min at 37°C. Cells were then washed with PBS, fixed in 4% paraformaldehyde, and permeabilized with 0.05% Triton-X at room temperature for 10 min. Fluorescence and DIC images of cellular morphology were obtained using a digital camera with a charge-coupled device image sensor on an Olympus FluoView™ FV1000 microscope equipped with a DIC channel. Images were acquired sequentially in all channels in order to avoid emission crosstalk.

### Immunoblotting

After treatment with WGA or inhibitors, cells were washed twice with 1 x PBS and harvested using RIPA cell lysis buffer (50 mM Tris, 150 mM NaCl, 1% NP-40, 0.5% sodium deoxycholate, 0.1% SDS) and 1 x protease inhibitor cocktail. Equal amounts of protein were subjected to 10%–15% SDS-PAGE and electrotransferred onto PVDF membranes (Millipore Corporation, Billerica, MA, USA). Membranes were blocked in 5% fat-free milk in Tris-buffered saline with 0.1% Tween 20 to prevent non-specific binding and incubated at room temperature for 1 h. The membranes were incubated with primary antibody at 4°C for 16 h and then incubated with the appropriate secondary antibodies at room temperature for 1 h. Protein signals were detected by chemiluminescence using the horseradish peroxidase substrate Luminol (Millipore) and visualized with a UVP imaging system (UVP, Upland, CA, USA).

### Caspase assays

Caspase-3, -7, and -9 activity in WGA-treated cells was measured using the Caspase-Glo Assay kit (Promega). Briefly, the pro-luminescent substrate containing the amino acid sequences DEVD, LETD, or LEHD was cleaved by caspase-3, -7, or -9, respectively. After caspase cleavage, a substrate for luciferase (aminoluciferin) was released, resulting in a luminescent signal. Cells were seeded into 96-well plates at a density of 5 x 10^3^ cells per well with different concentrations of WGA (0.05, 0.5, 5, and 15 μg/mL) and incubated for 24 h. An equal volume of Caspase-Glo reagent was added to each well and the cells incubated at room temperature for 1 h. Luminescence was measured using a luminometer plate reader.

### Knockdown of Alfy by RNA interference

HeLa cells were infected with Alfy siRNA lentiviral particles (sc-89191-v, Santa Cruz Biotechnology Inc., Santa Cruz, CA, USA) or control shRNA lentiviral particles (sc-108080). Briefly, HeLa cells were grown in 6-well plates to approximately 50% confluence, supplied with fresh medium containing Polybrene^®^ (5 μM), and transduced with shRNA lentiviral particles. Cells were then incubated for 24 h. Following the incubation, the medium containing the virus was removed and replaced with complete medium without Polybrene^®^. The cells were treated with puromycin (1 μM) 24–48 h later to select for puromycin-resistant clones.

### Effects of the translation inhibitor on WGA-induced cytoplasmic vacuolization

Cells were pretreated with CHX (25 μM) for 1.5 h and then treated with WGA at a concentration of 5.0 or 10 μg/mL for 24 h. The extent of cytoplasmic vacuolization was visualized by bright field microscopy.

### Statistical analysis

Statistical analysis was performed on the results from at least three independent experiments using the GraphPad Prism 5.0 software. Data were analyzed using Student’s *t*-tests and expressed as the mean ± SD. A P-value <0.05 was considered significant.
